# Detailed Study of the Correlation between Cross-Linking of Thick SU-8 and UV–NIR Optical Transmission/Photoluminescence Spectroscopy

**DOI:** 10.3390/polym15193866

**Published:** 2023-09-23

**Authors:** Abdullah Alharbi, Dhaifallah Almutairi, Hadba Hussain, Salman Alfihed

**Affiliations:** Microelectronics and Semiconductors Institute, King Abdulaziz City for Science and Technology (KACST), Riyadh 11442, Saudi Arabia

**Keywords:** SU-8 cross-linking, SU-8, photoluminescence, SU-8 optical transmission, SU-8 characterization, SU-8 thermal effects on cross-linking, SU-8 UV annealing effects on cross-linking

## Abstract

SU-8 polymers are promising materials for various applications due to their low cost, excellent thermal stability, and outstanding mechanical properties. Cross-linking of SU-8 is a crucial process that determines the properties of the materials. This study investigates the effect of cross-linking of free-standing SU-8 films on optical transmission and PL emission under various curing conditions. Our findings show that an increase in the cross-linking density reduces optical transmission and causes a red shift of the PL emission band peaks. By directly measuring the optical response of the isolated SU-8, we remove any uncertainty due to the substrate’s presence. Moreover, we show that optical transmission and PL spectroscopy are two non-distractive techniques that can be employed to monitor the curing of the SU-8. This finding enhances our understanding of the cross-linking process in SU-8 and paves the way to further enhance the properties of the SU-8 polymer for various electronics and optoelectronics applications.

## 1. Introduction

SU-8 is a negative-tone photoresist widely used in microfabrication [1], microfluidics [2], photonics [3,4], and microelectronics [5,6] applications [7]. It is known for its superior coating of greater than 1 mm, planarization, and processing properties, as well as its mechanical, thermal, and chemical stability [8]. The polymer curing process is induced via two methods, one, by adding chemicals such as polyamines to harden the epoxy resins, and the other method is curing in the absence of chemical additives such as through light exposure, heat treatment, and radiation [9]. The curing process of SU-8 is critical to its performance, and by controlling the curing process judiciously, it is possible to create a high-quality SU-8 layer that is suitable to meet the needs of the applications.

The cross-linking process is a useful method for various applications such as polymer gas separation membranes [10,11] and the formation of ZIF-8 filler particles [12,13,14]. In addition, finding the perfect degree of the cross-linking process changes the properties of materials. The curing process of SU-8 depends on various parameters, such as layer thickness, curing time, post-exposure baking (PEB) time, temperature degrees of heat treatment, and ultraviolet (UV) exposure dose [15,16]. When Kalaiselvi et al. [17] significantly investigated SU-8 cross-linking using Fourier-transform infrared spectroscopy (FTIR) for monitoring, the results showed that the rate of cross-linking declines after an interval of PEB time regardless of PEB temperature, as well as a connection between the alteration of the absorbance area of the peak at 914 cm^−1^ and the X-ray exposure dose. As a result, the indeclinable polymer structure obtained [18,19], as well as the optical transmission and photoluminescence (PL) properties, changed during the curing process and were altered based on the wavelength range [20].

SU-8 is transparent above 400 nm as well as at telecommunications wavelengths [21], exposed and unexposed, and the transmission of SU-8 is reduced with an increase in the hard-backing temperature [22]. In Ref. [23], the impact of UV exposure and heat treatment on the reflectance of SU-8 films with thicknesses from 10 µm to 157 µm was varied, showing that the polymer’s reflectance spectra are thickness dependent [23]. Furthermore, the SU-8 reflectance spectra increased and blue shifted with increasing scum thickness, leading to the pillars becoming efficiently shorter in the infrared (IR) range [24].

The cationic photopolymerization of an SU-8 photoresist was studied using the effect of UV light on PL spectroscopy [7]. The PL curve results showed that the cationic photopolymerization was induced in the presence of UVA light, and, in partial absence, by adding the carbon nanotubes to the SU-8, the SU-8 PL was quenched. However, the PL of SU-8 can be used as a sensing transduction parameter, as Eravuchira et al. [25] reported for the immunosensing field. The PL results showed an organized reduction based upon modification of its surface chemistry. Moreover, the PL results in [26] indicated the presence of an oxidation layer on the quantum dots by shifting the peaks to the blue side. In addition, a study of the optical features of curing SU-8 included Raman scattering, where its intensity changed by 1 wt% for the photoinitiators and induced the heating and cross-linking of photopolymers. The strongest changes in the Raman scattering results were placed in a narrow 895 cm^−1^ band [27].

In this work, we study the correlation between thick SU-8 cross-linking and optical transmission in the UV, visible, and near-infrared (NIR) ranges and the PL emission in the range from 400 nm to 800 nm. Our results improve our understanding of the SU-8 cross-linking process and can lay the foundation for continuing advancements to enhance SU-8’s properties for diverse applications.

## 2. Materials and Methods

The process of SU-8 polymer curing involves several steps, including coating, pre-baking, UV exposure, and post-exposure baking. In order to fabricate high-quality SU-8 structures, it is essential to cure the polymer to achieve the desired properties. Typically, in the process of curing, the sample is heated on a hotplate or in an oven and exposed to UV light to promote the cross-linking of SU-8 molecules, resulting in the formation of a strong and long-lasting layer. By curing SU-8 polymers, they are transformed from a liquid state to a solid material that exhibits well-defined shapes and features.

Thermal curing using a hotplate and oven is the most common method and requires systematic control of both the temperature and time to achieve optimal results. The curing temperature for SU-8 typically ranges from 65 °C to 95 °C, and the curing time can range from several minutes to several hours, depending on the thickness of the layer and the specific type or grade of SU-8 being used. Thicker layers of SU-8 generally require longer curing times and higher temperatures to ensure complete cross-linking and proper adhesion. It is worth noting that over-curing can cause the SU-8 to become brittle and crack, while under-curing can cause incomplete cross-linking and poor adhesion. Therefore, it is crucial to carefully select the appropriate curing method and parameters based on the specific application and desired properties of the SU-8 layer.

Here, we investigate how the optical properties of SU-8 are affected by various curing parameters. Thick SU-8 2050 films of an epoxy-based negative photoresist produced by Kayaku Advanced Materials, Westborough, MA, USA [28] were prepared by dispensing equal volumes of SU-8 polymer onto 50 mm diameter glass Petri dishes. The Petri dishes were used as substrates for the samples during the curing process. Once the SU-8 samples were cured, they were removed from the Petri dishes to obtain free-standing films, and the measured thicknesses of the samples were 2.4 ± 0.8 mm. Each film was then cut into four pieces for different measurements, including PL, transmission, and water swelling measurements. The samples were then characterized to obtain direct optical measurements. This method accurately measures the optical properties of free-standing cured SU-8 films. The measurement of the isolated films mitigates any potential ambiguity arising from the presence of the substrate.

We studied the effect of three curing parameters, post-exposure curing time, curing temperature, and UV exposure time. Post-exposure curing times of 30, 90, 120, and 150 min were investigated. Post-exposure curing temperatures of 80 °C, 100 °C, 110 °C, and 120 °C were investigated. UV exposure times of 60, 120, 180, 240, and 300 s were studied using a 360 nm UV lamp with a power density of 10.338 mW/cm^2^, which corresponds to a dose of 620 mJ/cm^2^ per min. The preparation parameters of the samples are summarized in Table 1.

In order to achieve uniform curing of the SU-8 material without the occurrence of cracks, a two-stage baking procedure was implemented for all specimens. In the initial phase, the polymer was subjected to a thermal treatment at a specific temperature of 65 °C for 30 min. The first baking procedure has a vital role in reducing stress and preventing the occurrence of cracks in SU-8 during the subsequent curing process, a commonly encountered problem [29]. The temperature was elevated to the intended level following the initial phase to finalize the curing procedure. This methodology not only guarantees consistent curing of the SU-8 but also serves to augment its mechanical and chemical characteristics, which are vital for achieving the outcome. Hence, adhering to a two-stage baking procedure is crucial for obtaining the most favorable outcomes when handling SU-8.

## 3. Results and Discussion

This section presents the results of a comprehensive study that examined the relationship between SU-8 cross-linking and optical transmission alongside photoluminescence spectroscopy. Firstly, we discuss the fundamental connection between cross-linking and optical transmission. Next, we delve into the correlation between photoluminescence spectroscopy and cross-linking. Finally, we scrutinize the link between swelling behavior in water and cross-linking.

### 3.1. Transmission Spectrum

The optical transmission of SU-8 in the UV–NIR range can be affected by its thickness, exposure dose, and curing temperature. In this subsection, we present the transmission spectra of SU-8 films as a function of thermal curing time, curing temperature, and UV exposure time.

**Figure 1:**
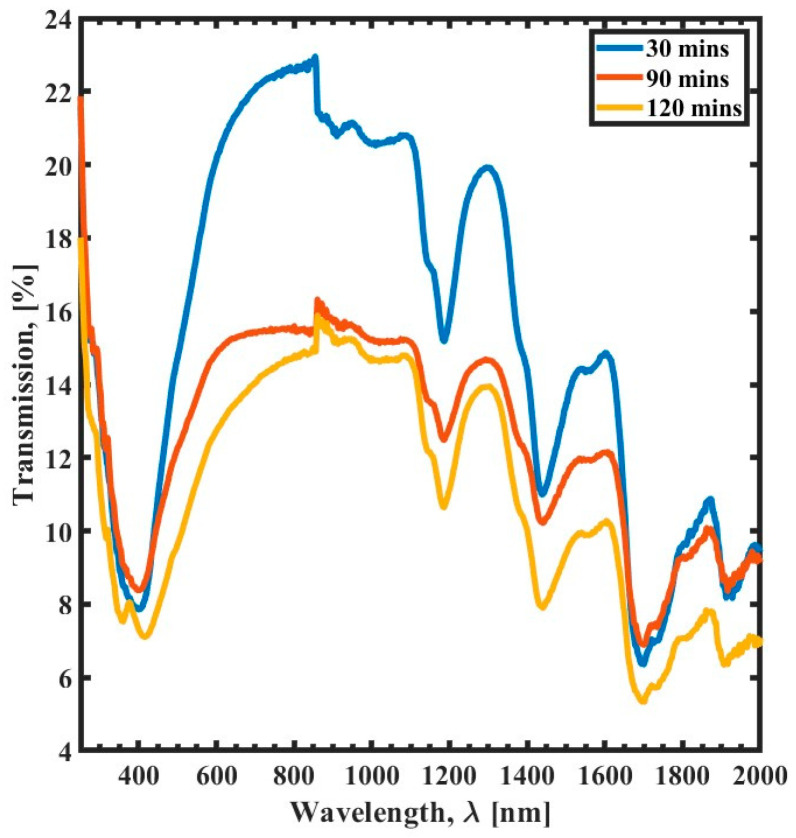
Transmission spectrum (300 nm to 2000 nm) of the curing time variation as a function of wavelength (λ) for 30 min curing time (blue), 90 min curing time (red), and 120 min curing time (yellow).

The transmission spectrum of SU-8 films, a common photoresist polymer used in microfabrication processes, changes as a function of thermal curing time, as shown in Figure 1. The spectrum exhibits five distinct absorption peaks corresponding to different chemical bonds in the polymer. One of these peaks occurs in the UV range (300–400 nm), while the other four occur in the NIR range (700–900 nm). The maximum transmission of SU-8 film is 22% at 840 nm when cured for 30 min. However, as the curing time increases, the transmission decreases due to the cross-linking of the polymer chains. Cross-linking is a process in which the polymer chains are linked, forming a more rigid structure. This rigid structure is less transparent than the uncured polymer, resulting in a decrease in transmission.

Curing the SU-8 film for 90 min reduces the transmission by 6%, but the peaks do not shift. This suggests that the cross-linking of the polymer occurs uniformly throughout the film. This uniformity is critical for applications where the optical properties of the SU-8 film are essential—curing the SU-8 film for longer than 90 min results in only a slight decrease in transmission (less than 2%). This decrease is likely due to the degradation of the polymer chains, which results in a loss of transparency.

The lack of a shift in the peaks with increasing curing time and the uniform cross-linking throughout the film demonstrates the consistency of the SU-8 film. This consistency is crucial for fabricating microdevices, where a precise and uniform pattern is necessary. The optical properties of the SU-8 film are also essential for the performance of microdevices, such as photonic crystals, waveguides, and biosensors. Therefore, understanding the transmission spectrum of SU-8 films as a function of thermal curing time is crucial for successfully fabricating microdevices.

**Figure 2:**
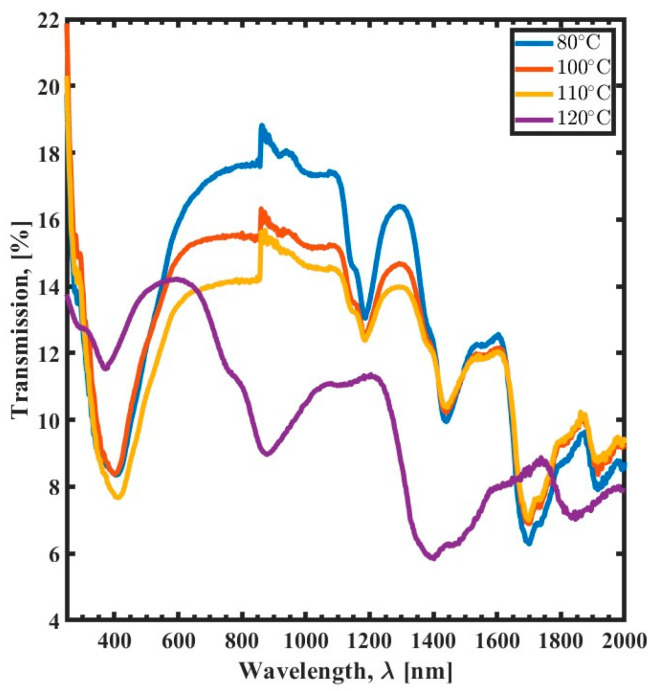
Transmission spectrum (300 nm to 2000 nm) of the thermal curing temperature variation as a function of wavelength (λ) for 80 °C (blue), 100 °C (red), 110 °C (yellow), and 120 °C (purple).

Figure 2 depicts the transmission spectrum of SU-8 films in relation to curing temperature. The spectrum exhibits five absorption peaks, one in the UV range and four in the NIR range, as illustrated in Figure 1. However, when cured at 120 °C, only four absorption resonances are visible since the higher temperature suppresses the fifth peak in the NIR range. The optimal transmission is achieved at 80 °C curing temperature, with a peak transmission of 18% at a wavelength of 860 nm. Increasing the annealing temperature to 100 °C results in a reduction of approximately 2% in the transmission spectrum. When the temperature is raised to 120 °C, there is a 5% decrease in transmission.

The decline in transmission with increasing curing temperature is associated with the cross-linking of the SU-8 polymer. Cross-linking occurs when polymer chains bond to form a more rigid structure, which is less transparent than the uncured polymer and decreases transmission. The suppression of the fifth peak in the NIR range when the curing temperature is set to 120 °C is most likely due to the degradation of the polymer chains. Degradation occurs when the polymer chains break down, resulting in a loss of transparency.

**Figure 3:**
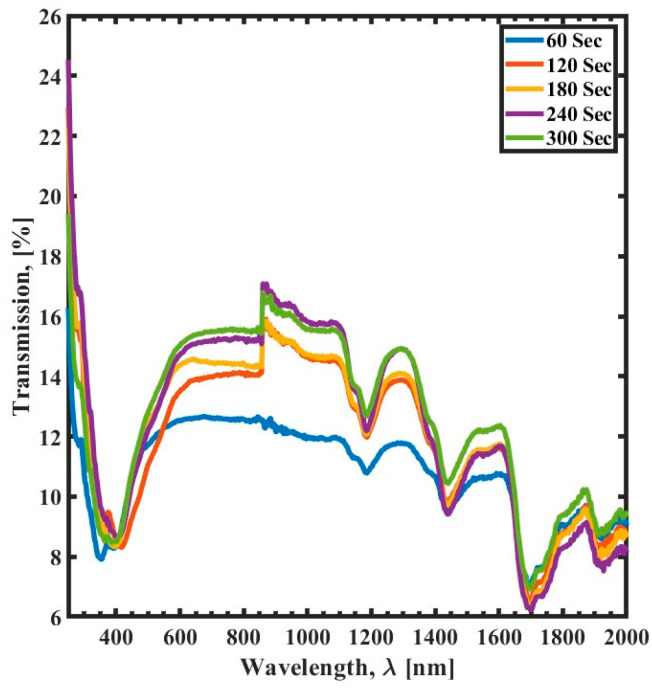
Transmission spectrum (300 nm to 2000 nm) of the UV exposure time variation as a function of wavelength (λ) for 60 s (blue), 120 s (red), 180 s (yellow), 240 s (purple), and 300 s (green).

Figure 3 illustrates the transmission spectrum of SU-8 films as a function of UV exposure time. The transmission spectrum exhibits a distinct absorption resonance at a specific wavelength. However, the transmission decreases with decreasing UV exposure time. The highest transmission occurs when the UV exposure time is set to 300 s, with a transmission value of 17%. A relatively similar value is observed when the UV curing time is 240 s. However, when the UV curing time is reduced to 60 s, the maximum transmission spectrum is reduced by 4% compared to the transmission spectrum when the curing time is set to 300 s. The decrease in transmission with decreasing UV exposure time is because the cross-linking of the SU-8 polymer is not as complete. When the SU-8 is exposed to UV light, the polymer chains are linked, forming a more rigid structure. This rigid structure is less transparent than the uncured polymer, resulting in a decrease in transmission. The degree of cross-linking is directly related to the UV exposure time, and shorter exposure times result in a less complete cross-linking process and a lower transmission.

The cationic photopolymerization process can occur when SU-8 is exposed to UV light, resulting in the formation of a cross-linked polymer network and a change in the optical transmission of the layer. The thickness of the polymer network layer is determined by the amount or the length of time of UV exposure, which impacts the optical transmission of the SU-8. The results in Figure 3 suggest that the optimal UV exposure time for SU-8 films is 300 s. This is because the transmission spectrum is maximized at this time, and the decrease in transmission with decreasing exposure time is minimal. The graph also shows that a curing time of 240 s results in a relatively similar transmission value to that achieved with 300 s of exposure. However, a curing time of 60 s results in a significant reduction in transmission compared to the optimal 300 s of exposure.

The results show that the transmission spectrum of SU-8 films is affected by the curing parameters of annealing time, temperature, and UV exposure time. The optical transmission of SU-8 films decreases with increasing thermal curing time or temperature. This is because thermal curing strengthens the formation of cross-links between polymer chains. However, when increasing the UV exposure time, it is observed that the transmission spectrum increases. This leads to the conclusion that prolonged exposure to the UV reduces the formation of the cross-links between polymer chains, which is counterintuitive. The cross-linking rate increases as the temperature increases, leading to reduced transmission. Additionally, trapped moisture in the SU-8 layer causes a significant reduction in transmission and results in absorption peaks [30]. This is because the moisture molecules absorb light, which reduces the amount of light that can pass through the film.

### 3.2. Photoluminescence Spectroscopy

The result of the photoluminescence properties of the polymer was studied under various curing processes. The excitation wavelength was 400 nm, and PL spectra were recorded from 420 nm to 780 nm to study the variations of the two emission bands with maxima at ∼425 nm and ∼555 nm.

**Figure 4:**
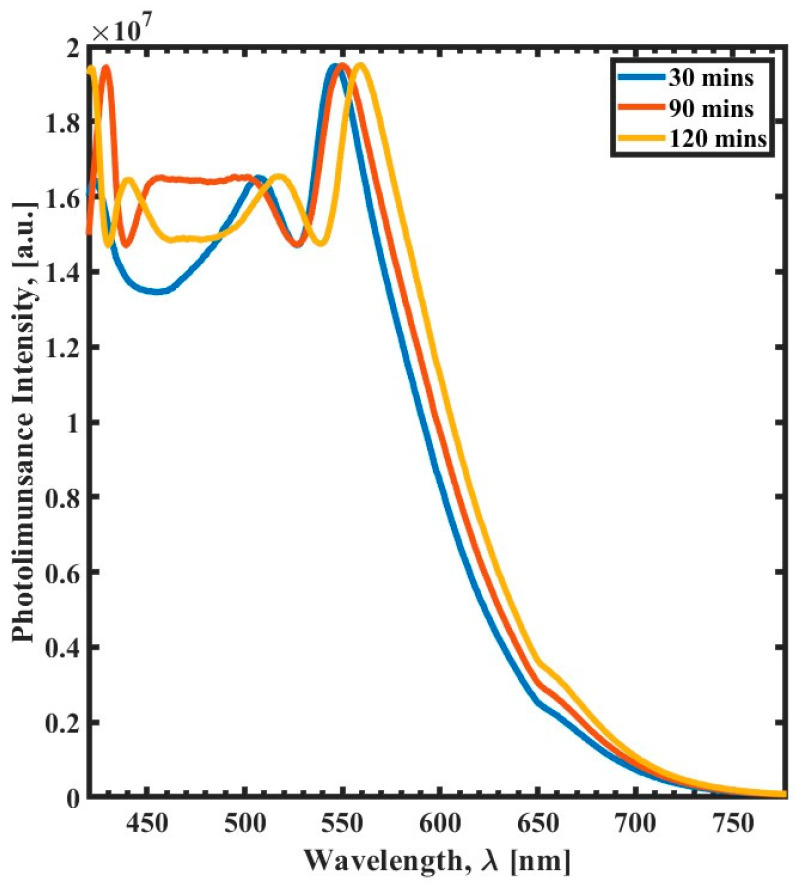
Photoluminescence spectra (420 nm to 780 nm) of the curing temperature variation as a function of wavelength (λ) for 30 min curing time (blue), 90 min curing time (red), and 120 min curing time (yellow).

Figure 4 shows the PL spectra of samples cured using different curing times. The figure demonstrates that increasing the duration of the post-exposure curing process increases the difference between the two peaks by shifting them in opposite directions. The shift is more pronounced for the emission bands with maxima at ∼555 nm, where the peak exhibits a red shift with an increase in the post-exposure curing time from 30 min to 120 min. Moreover, the film cured for a 120 min spectrum exhibits four distinct PL peaks. The difference between the two emission bands’ maxima and the number of distinct PL peaks might be used to monitor the curing process.

**Figure 5:**
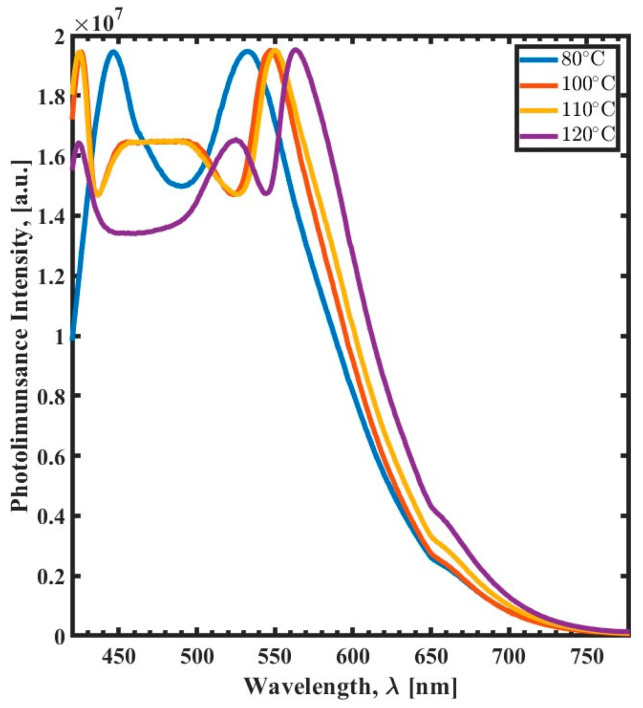
Photoluminescence spectra (420 nm to 780 nm) of the curing temperature variation as a function of wavelength (λ) for 80 °C (blue), 100 °C (red), 110 °C (yellow), and 120 °C (purple).

The effect of the post-exposure curing temperature is illustrated in Figure 5. The same observation as depicted in Figure 2 shows that the difference between the two emission bands increases with the temperature. Additionally, the PL peaks exhibit a red shift when increasing the curing temperature from 80 °C to 120 °C. The number of distinct PL peaks increases for the cured film at a curing temperature of 120 °C. However, the full width at the half maximum of the PL spectrum of the SU-8 is reciprocal to the curing temperature. The film that is cured at 80 °C is more comprehensive than the others due to the overlap of multiple PL emission peaks.

**Figure 6:**
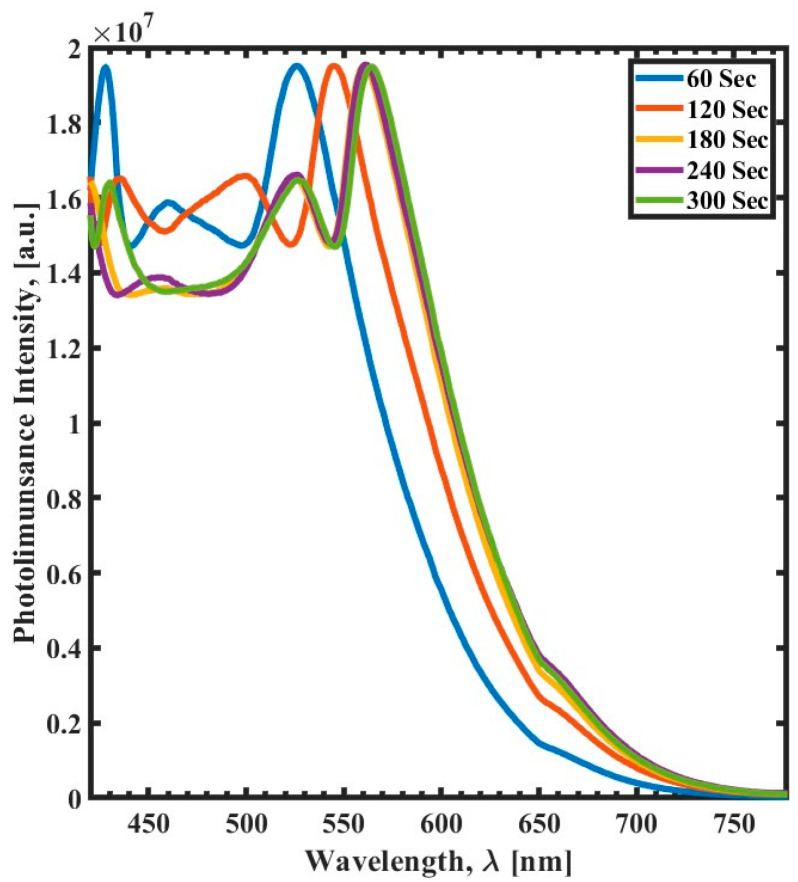
Photoluminescence spectra (420 nm to 780 nm) of the UV exposure time variation as a function of wavelength (λ) for 60 s (blue), 120 s (red), 180 s (yellow), 240 s (purple), and 300 s (green).

We also investigated the influence of the UV exposure time of SU-8 on the PL spectra, as shown in Figure 6. The PL spectra reveal that the PL emission band maximum at 550 nm exhibits a clear blue shift to 525 nm and 544 nm for samples cured at 60 s and 120 s, respectively—the effect of UV exposure time becomes negligible after 180 s. In addition, UV samples cured for only 60 s show an emission peak at 427 nm. The behavior of PL spectra here agrees with the results reported by Baibarac et al. in their study of the UV effect on SU-8 cationic photopolymerization, in which two prominent peaks appeared in the UV and red ranges [7].

These measurements reveal that the PL measurement method is a powerful tool for monitoring the SU-8 curing process. From all the previous three curing parameters, we can see the position of the PL emission band maximum at ~550 nm for SU-8 films changes as a function of the curing process.

### 3.3. Swelling Behavior in Water

Cross-linking occurs in SU-8 during curing via acid-initiated cationic polymerization after exposure to UV radiation and heating [31,32]. By adjusting the UV exposure energy, the amount of cross-linking inside an SU-8 film can be altered [31]. SU-8 is a highly cross-linked polymer, and the water molecules penetrate into the polymer to fill the free volume within the polymer [33]. This leads to changes in the microstructure and the physical properties of the material. Ref. [34] found that SU-8 is prone to swelling, indicating water absorption into the structure, and calculated a water diffusion coefficient in SU-8. In their study, they provide an illustration of the chemical composition of the monomer SU-8, which consists of eight epoxy groups [34]. This leads to changes in the microstructure and the physical properties of the material. The SU-8 water swelling can be affected by the thickness of the film and the temperature [35]. The study focused on the cross-linking density of SU-8 using the polymer swelling method. Previous studies have shown that polymers with lower levels of cross-linking have a greater tendency to absorb water. This absorption leads to an increase in the sample’s weight and swelling [36,37,38]. In this study, we utilized the swelling behavior in water by measuring the amount of water absorbed. The quantity of water absorbed was determined by monitoring the change in weight of the SU-8 film upon exposure to water at room temperature.

**Figure 7:**
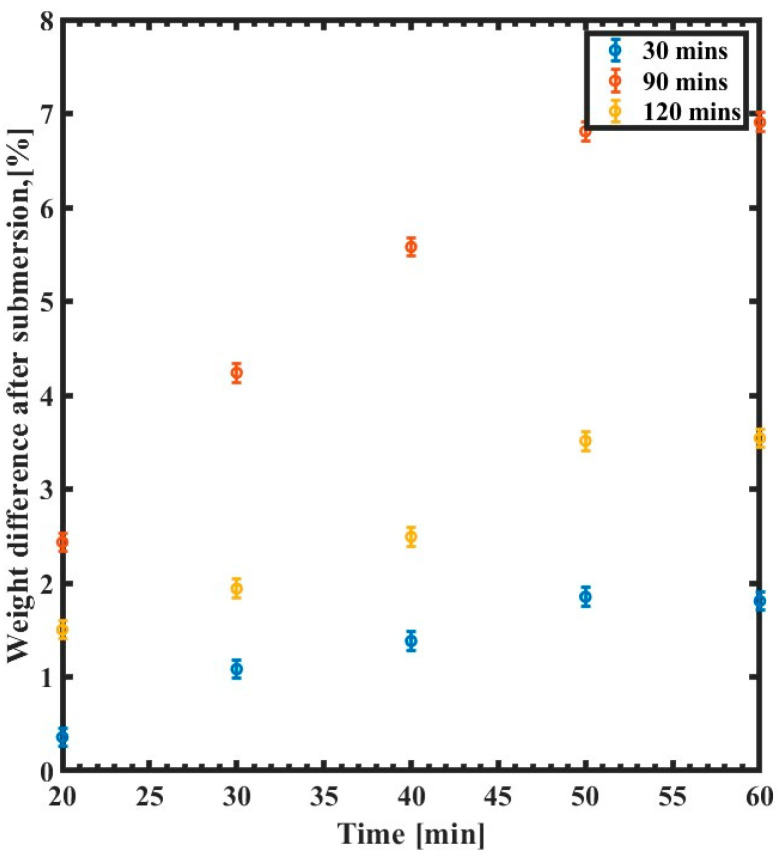
Weight difference of the SU-8 curing time variation as a function of immersion time (min) for 30 min curing time (blue), 90 min curing time (red), and 120 min curing time (yellow).

Figure 7 illustrates the relative weight changes of SU-8 films as a function of water immersion time for different annealing times. The weight increases during the first 50 min in all samples and then reaches a plateau. Such behavior manifests itself as a saturation of water contents within the polymer, which appears after 50 min for the tested thick SU-8 samples. Samples cured for only 30 min swell around four times more than those annealed for 120 min. The weight of the 30 min sample increases by 7%, while the change in the weight is less than 2% for the 2 h sample. The same behavior is observed for the sample baked for 90 min, where the weight change is more obvious during the first 50 min, reaching 3.5%, then becomes more stable as the SU-8 becomes saturated.

**Figure 8:**
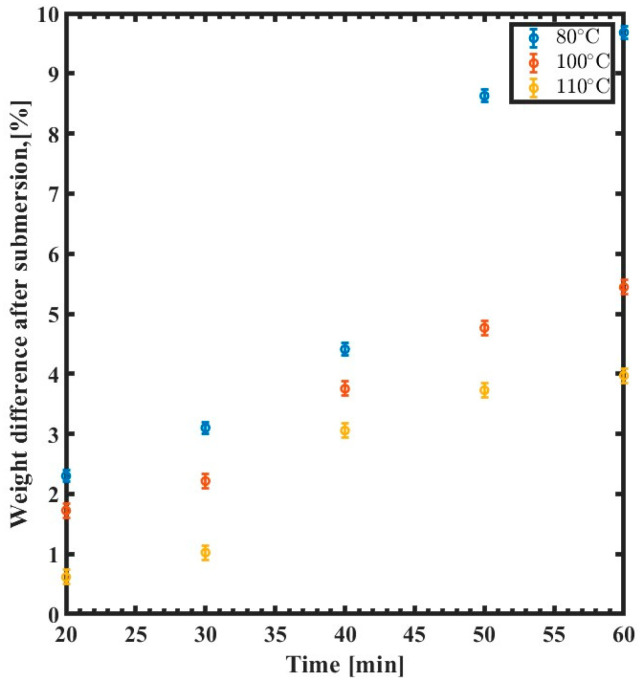
Weight difference of the SU-8 curing temperature variation as a function of immersion time (min) for 80 °C (blue), 100 °C (red), and 110 °C (yellow).

Figure 8 displays the difference in SU-8 in relation to immersion time as a function of the baking temperature. The weight of the sample that is annealed at 80 °C increases by ~10%, while samples that are cured at higher temperature exhibit less of a change in weight. For example, the weight of the sample that is annealed at 110 °C rises by less than 2%.

**Figure 9:**
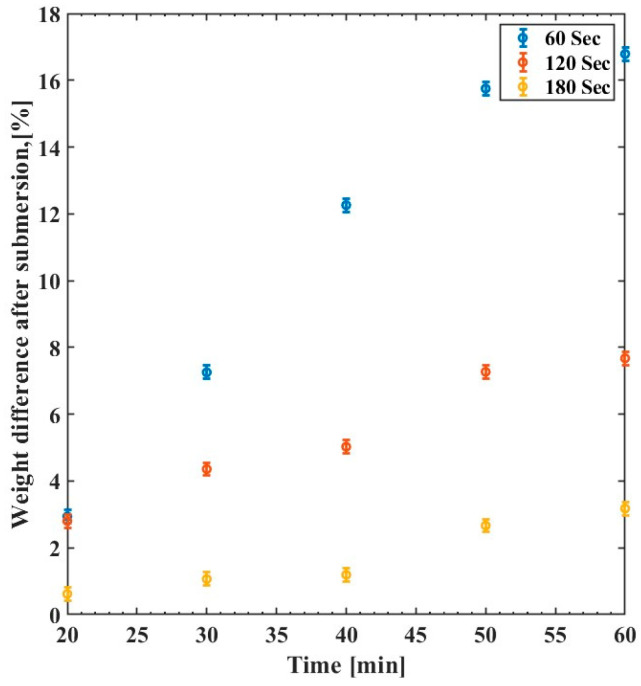
Weight difference of the SU-8 UV exposure time variation as a function of immersion time (min) for 60 s (blue), 120 s (red), and 180 s (yellow).

The degree of swelling in SU-8 can also be affected by UV exposure. The UV exposure can increase the degree of cross-linking in the polymer matrix and ultimately reduce its swelling, as shown in Figure 9. The change in sample weight increases rapidly for the first 50 min, then the difference is negligible. The weight of the samples cured for only 60 s and 120 s increases drastically by ~17% and ~8%, respectively. At the same time, the changes are much less for the samples that are cured for 3 min or more. Interestingly, the results align with the PL measurement reported in Figure 6, which indicates that the SU-8 films are well cross-linked upon UV exposure for 3 min (~1860 mJ/cm^2^). A summary of the SU-8 weight measurements due to water swelling is shown in Table 2.

Overall, the water swelling can be controlled by adjusting the curing conditions and post-curing treatments of the SU-8. For example, increasing the UV exposure, post-bake time, or curing temperature can reduce the amount of residual solvent in the polymer matrix and decrease the swelling. It is worth noting that the obtained water swelling findings are correlated with the obtained optical transmission results, since increasing the cross-linking (as proven by water swelling) is correlated with increasing the optical transmission (as proven by the UV–NIR transmission spectrum).

## 4. Conclusions

In this work, a variety of cross-linking conditions of SU-8 (including curing time, curing temperature, and UV exposure time) were investigated with respect to optical transmission/photoluminescence spectroscopy. The findings presented that the cross-linking of SU-8 plays a crucial role in determining its optical transmission and photoluminescence response. This study concretely focused on the correlation between the polymer cross-linking and the photoluminescence and optical transmission of SU-8 films. The findings revealed that the polymer cross-linking is inversely proportional to the photoluminescence emission band wavelength at ~550 nm and optical transmission. The combination of optical transmission and PL spectroscopy can be used to gain a deeper understanding of the optical properties of SU-8. This work’s findings can lay a foundation to optimize the design and fabrication of SU-8-based optical components and systems.

## Figures and Tables

**Table 1:** Summary of sample preparation parameters.

Sample Group	Temp. 1 (°C)	Temp. 1 Time (min)	UV Exposure (s)	Temp. 2 (°C)	Temp. 2 Time (min)
Group 1: cross-linking as a function of curing time	90	60	240	90	30
	90	60	240	90	90
	90	60	240	90	120
Group 2: cross-linking as a function of curing temperature	90	60	240	80	60
	90	60	240	100	60
	90	60	240	110	60
	90	60	240	120	60
Group 3: cross-linking as a function of UV exposure time	90	60	60	90	60
	90	60	120	90	60
	90	60	180	90	60
	90	60	240	90	60
	90	60	300	90	60

**Table 2:** Summary of the SU-8 weight measurements due to water swelling.

Weight Change Percentage as a Function of Time	Thermal Annealing Time (min)			Temperature (°C)			UV Curing Time (s)		
	30	90	120	80	100	110	60	120	180
20 min	2.434	1.502	0.358	2.299	1.723	0.614	2.939	2.80	0.616
30 min	4.239	1.939	1.083	3.100	2.215	1.025	7.254	4.357	1.063
40 min	5.579	2.493	1.382	4.408	3.751	3.055	12.262	5.026	1.192
50 min	6.810	3.514	1.853	8.626	4.765	3.726	15.750	7.269	2.665
60 min	6.906	3.542	1.808	9.681	5.441	3.968	16.787	7.675	3.179

## Data Availability

The data used to support the findings of this study are available from the corresponding author upon reasonable request.
